# A Standardised Approach to the Biomechanical Evaluation of Tracheal Grafts

**DOI:** 10.3390/biom11101461

**Published:** 2021-10-05

**Authors:** Néstor J. Martínez-Hernández, Jorge Mas-Estellés, Lara Milián-Medina, Cristina Martínez-Ramos, José Cerón-Navarro, José Galbis-Caravajal, Amparo Roig-Bataller, Manuel Mata-Roig

**Affiliations:** 1 Thoracic Surgery Department, Hospital Universitari de la Ribera, 46600 Valencia, Spain; galbis_joscar@gva.es (J.G.-C.); frude_2@hotmail.com (A.R.-B.); 2 Biomaterials Center, Universitat Politècnica de València, 46022 Valencia, Spain; jmas@fis.upv.es (J.M.-E.); crimarr2@upvnet.upv.es (C.M.-R.); 3 Pathology Department, Medicine and Odontology Faculty, Universitat de València, 46010 Valencia, Spain; lara.milian@uv.es (L.M.-M.); manuel.mata@uv.es (M.M.-R.); 4 Thoracic Surgery Department, Hospital Universitari i Politècnic la Fe, 46026 Valencia, Spain; ceron_jos@gva.es; 5 Networking Research Center on Respiratory Diseases (CIBERER), ISCIII, 28029 Madrid, Spain

**Keywords:** airway, bioengineering, tissue engineering, trachea, biomechanics, transplantation

## Abstract

The ideal tracheal substitute must have biomechanical properties comparable to the native trachea, but currently there is no standardised approach to evaluating these properties. Here we propose a novel method for evaluating and comparing the properties of tracheal substitutes, thus systematising both measurement and data curation. This system was tested by comparing native rabbit tracheas to frozen and decellularised specimens and determining the histological characteristics of those specimens. We performed radial compression tests on the anteroposterior tracheal axis and longitudinal axial tensile tests with the specimens anastomosed to the jaw connected to a measuring system. All calculations and results were adjusted according to tracheal size, always using variables relative to the tracheal dimensions, thus permitting comparison of different sized organs. The biomechanical properties of the decellularised specimens were only slightly reduced compared to controls and significant in regard to the maximum stress withstood in the longitudinal axis (−0.246 MPa CI [−0.248, −0.145] MPa) and the energy stored per volume unit (−0.124 mJ·mm^−3^ CI [−0.195, −0.055] mJ·mm^−3^). The proposed method is suitable for the systematic characterisation of the biomechanical properties of different tracheal substitutes, regardless of the size or nature of the substitute, thus allowing for direct comparisons.

## 1. Introduction

A wide range of conditions—including malignant tumours, benign stenosis secondary to trauma, as well as congenital, inflammatory, idiopathic, or iatrogenic causes—can cause local airway obstruction [1]. Regardless of the specific aetiology, airway obstruction negatively impacts quality of life and may even be life-threatening. In these cases, the involved tracheal segment must be resected to resolve the condition.

The gold standard treatment for both benign and malignant stenosis is the surgical removal of the affected area followed by reanastomosis [2]. However, due to the unique biomechanical and anatomic characteristics of the trachea, the maximum resection size is approximately 4.5 cm (7.2 rings) [2,3,4]. Reanastomosis of the trachea is a highly complex technique and, in many cases, reanastomosis is not feasible due to the quantity of tissue involved, which may preclude a non-tension anastomosis. Consequently, many tracheal patients cannot be offered curative treatment [5].

The trachea is an organ comprised of C-shaped rings made of hyaline cartilage with inner mucosa and outer connective tissue and smooth muscle on the posterior side [6]. Numerous tracheal substitutes have been developed in an effort to offer a solution to patients in whom conventional therapy fails. Autogenic and artificial or biological allogenic substitutes have been used, but with limited success (fewer than 20 successful implants worldwide) [7,8]. The ideal tracheal substitute should retain the biomechanical properties of the native trachea in both the longitudinal and transversal axes [9]. Although several different strategies have been proposed to evaluate the biomechanical properties of tracheal substitutes, no standardised approach has yet been developed to evaluate and compare these substitutes.

The focus of most currently available protocols is on the external diameter of the trachea, even though the inner diameter is the clinically relevant one. Moreover, there is wide heterogeneity in how tensile tests are performed (e.g., between hooks [10], clamps [11,12], etc.), which highlights the need for greater standardisation. Similarly, the statistical approach to data analysis differs from study to study. Besides, the study parameters (e.g., force, elongation, compression, etc.) are often not described in relation to the size (length, diameter) of the replacement [13,14], thus making it impossible to accurately compare substitutes of different lengths. Some studies have also used arbitrary approaches (e.g., visual calculation of Young’s modulus [11,15]) to evaluate the data while other studies have failed to assess key parameters such as maximal stress and strain, energy stored per unit of trachea volume (tensile tests), and stiffness or energy stored per unit of trachea surface (radial compression tests) [11,15,16]. In short, the studies performed to date have used highly heterogenous methods to determine the biomechanical properties of tracheal substitutes. As these examples provided above indicate, there is a clear lack of standardised methods to compare the biomechanical properties of tracheal replacements.

A proper tracheal substitute must maintain the biomechanical characteristics of the native trachea [17], but at present there is no standard method of determining those characteristics. In this context, the aim of the present study was to develop a valid, standardised protocol for the analysis of the biomechanical properties of all types of tracheal substitutes used for airway replacement. This study is based on the proposal made by Jones and colleagues regarding a standard method for studying the biomechanical properties in rabbit tracheae [15].

## 2. Materials and Methods

In this study, we tested a novel systematic method for evaluating and comparing the properties of tracheal substitutes. We tested this system by comparing native rabbit tracheas (controls) to frozen decellularised specimens.

### 2.1. Ethics Approval and Animal Research

This study adhered to the European directive (20170/63/EU) for the care and use of laboratory animals. The study protocol was approved by the Ethics Committee of the University of Valencia (Law 86/609/EEC and 214/1997 and Code 2018/VSC/PEA/0122 Type 2 of the Government of Valencia, Spain).

### 2.2. Tracheal Specimens

Control tracheas were obtained from eight white male New Zealand rabbits (*Oryctolagus cuniculus*), ranging in weight from 3.5 to 4.1 kg. The animals were euthanised with an intravenous bolus of sodium pentobarbital (Vetoquinol; Madrid, Spain). The tracheas, from the cricoid cartilage to the carina, were extracted through a central longitudinal cervicotomy and transported in sterile containers containing phosphate buffered saline (PBS; Sigma Chemicals, Barcelona, Spain).

### 2.3. Tracheal Decellularisation

**Figure 1:**
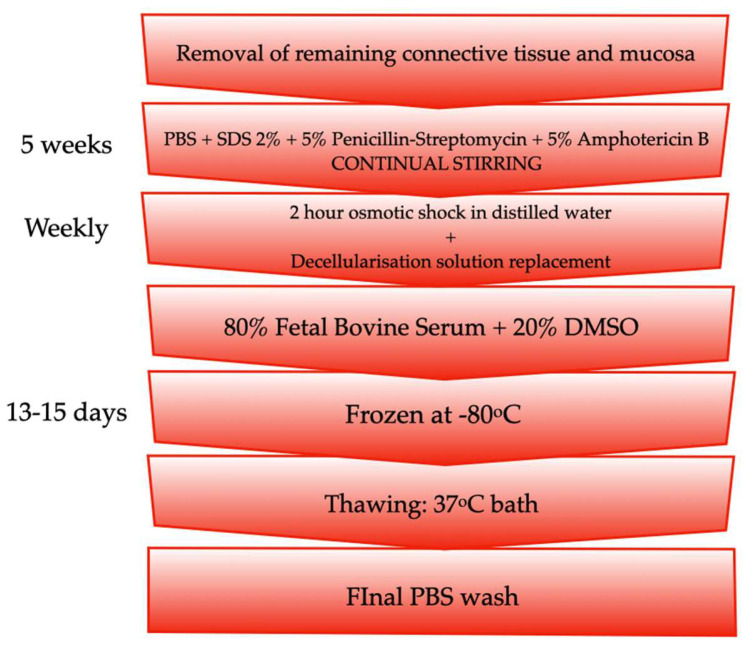
Flowchart of the decellularisation process.

The decellularisation technique has been fully described elsewhere [18]. Briefly, the tracheas were sectioned into specimens measuring 2 cm in length. All remaining connective tissue and mucosa were removed [19]. The specimens were then submerged in a PBS solution containing 2% sodium dodecyl sulfate (SDS; Sigma-Aldrich, San Luis, MO, USA), penicillin-streptomycin 5% and amphotericin B 5% (both obtained from Gibco^TM^ Thermo Fisher Scientific; Waltham, MA, USA) for 5 weeks under continual stirring. The decellularisation solution was replaced weekly after a 2-h osmotic shock in distilled water. The specimens were then frozen in a mixture of 80% fetal bovine serum (GE Healthcare Hyclone; Madrid, Spain) and 20% dimethyl sulfoxide (DMSO; Sigma-Aldrich; San Luis, MO, USA) at −80 °C until use. Defrosting was carried out in a 37 °C bath followed by a final wash with PBS (Figure 1). Decellularisation was evaluated by DAPI staining. DNA concentration was estimated through spectrophotometry (measuring absorbance at 260/280), using the Nanodrop spectrophotometer (Isogen Life Science. Utrech, Netherlands). The size of the extracted DNA was evaluated through chromatography using the Agilent bioanalyzer (Agilent, Santa Clara, CA, USA).

### 2.4. Biomechanical Study

To perform the biomechanical characterisation, axial tensile and radial compression tests were carried out to measure tracheal resistance to both longitudinal and transversal forces.

A Vernier caliper was used to measure tracheal length, wall thickness, and external diameter. Mean values were calculated from three random measurements of each variable. In the radial compression tests, the anteroposterior diameter was calculated by detecting the point at which the plate came into contact with the specimen. All tests were performed at room temperature.

#### 2.4.1. Tensile Tests

Tensile tests were performed on a desktop Universal Testing Machine (UTM) using Adamel Lhomargy DY34 (Testing Machines; Veenendaal, The Netherlands) displacement control, equipped with a 100 N load cell with TestWorks 4 software (MTS Systems Corporation; Eden Prairie, MN, USA), (0.1 N force resolution, 0.001 mm of position, and 0.1 s). Data were recorded every 0.4 s and exported to Microsoft Excel software for analysis (Microsoft Excel for Mac, v.16.23, Redmond, WA, USA).

**Figure 2:**
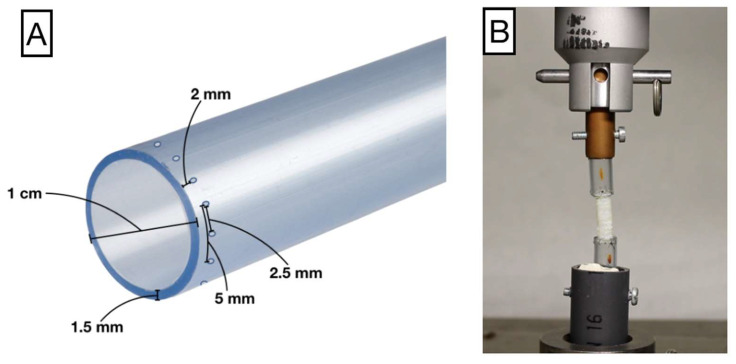
(**A**) Jaw model for trachea suture and stress analysis. (**B**) Trachea sutured to the jaw with a continuous suture. The jaws are attached to an adapter connected with the UTS.

Jaws adapted to the mean caliber of the tracheas were constructed from pure monolayer, non-toxic crystal polyvinyl chloride (PVC) hollow tubes (Cristallo Extra; FITT, Sandrigo, Italy) with an external diameter of 1 cm, and a wall thickness of 1.5 mm. To prevent measurement bias due to the presence of sutures, 12 preformed holes for the termino-terminal suture were punched 2 mm from the edge of the jaws separated by a distance of 2.5 mm (Figure 2).

The PVC glass tubes were attached to the rabbit trachea by termino-terminal anastomosis with a continuous 6–0 nylon monofilament suture (Monosoft^TM^; Covidien; Mansfield, MA, USA) through alternate preformed holes (every 5 mm) located 2 mm from the edge of the trachea.

All of the pieces were stretched at a displacement rate of 5.0 mm·min^−1^, with data recording every 0.4 s. Stress (σ) was calculated in megapascal (MPa) for each measured force value (*F* in N), as follows: *σ* = *F*/*A*, where A is the area (mm^2^) of the tracheal section calculated according to the formula for a thin crown: *A* = *2πRe*, where *R* is the outer radius and e the thickness of the piece in mm.

**Figure 3:**
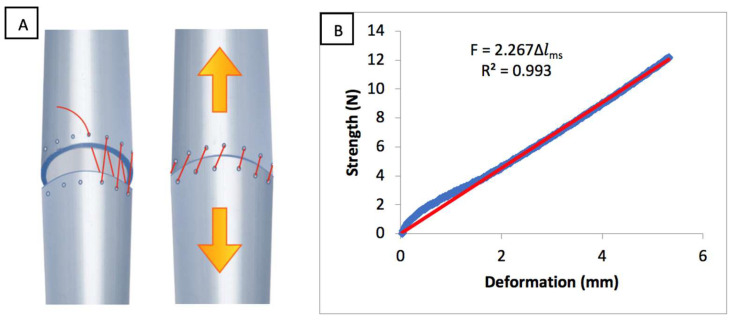
(**A**) Assembly of the two anastomosed jaws by the same technique used for the tracheas. (**B**) Force–deformation curve of the jaw-suture set (blue line). The trend line (in red) has a slope of 2.267 N·mm^−1^ (*F*: Force, ∆*l_ms_*: suture and jaws deformation, *R^2^*: correlation coefficient).

Prior to starting the test, the inherent deformation of the jaws and sutures was calculated to subtract this value from the final measurement. Both jaws were sutured together with the same suture and technique described above. The tensile test was then performed on this assembly. A force/deformation graph was obtained. The linear regression equation was determined by the least squares method; *F* = 2.267 ∆*l_js_* (slope 2.267 N·mm^−1^ and R^2^ = 0.993), with ∆*l_js_* representing the jaw and suture deformation in mm (Figure 3).

The first point at which force was detected on the trachea was taken as the initial position (origin). From this point, the displacement (∆*l*_0_, in mm) was measured by the UTS. The ∆*l*_js_ was obtained using the aforementioned equation. ∆*l*, the deformation of the trachea on applying a force *F*, was obtained after subtracting it; ∆*l* = ∆*l*_0_ − ∆*l_js_*. Strain (*ε*, unitless) was calculated by dividing the deformation at each point by the initial length of the piece (*L*_0_); *ε* = ∆*l*/*L*_0_.

The objective of any research on tracheal substitutes is to determine whether the substitute will maintain its integrity in a future implant. Since any small tear produces a fistula with its associated complications, such as infection of the area and graft failure [20,21], the first point at which any breakage occurs is defined as the limit of resistance. This was detected by the UTS as a reduction in the stress. However, as there were some reductions due to tissue or suture repositioning that did not end in rupture, the break point was defined as the point where stress dropped by >1% without recovery (i.e., a decrease >1% that was maintained or increased) at the next two data points (0.8 s). Stress and strain were obtained from this point on, considered as maximal breaking point values (*σ_max_* and *ε_max_*).

To determine the energy stored per unit of trachea volume (*W*/*Vol*), the area between the curve and the horizontal axis was calculated as its integral by Riemann sum with approximation at the midpoint. The result was obtained in mJ·mm^−3^.

**Figure 4:**
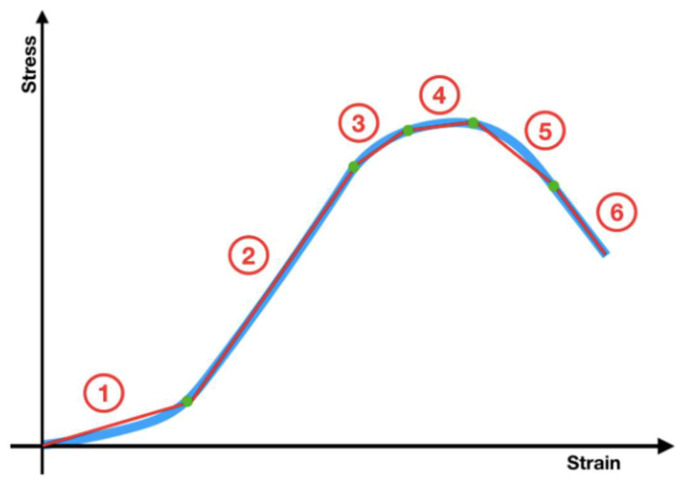
Stress–strain curve defining the segmented linear regression lines into which the curve can be broken down. Number 2 shows the slope of the last segment before it reached the proportional limit compared to the previous one.

The Young’s modulus (*E*) for each trachea (in MPa) was computed by applying segmented linear regression models comparing the slope of the last linear segment of the curve before reaching the proportional limit compared to the previous one (Figure 4).

#### 2.4.2. Radial Compression Test

Radial compression tests were performed on a desktop Microtest UTM (Microtest; Madrid, Spain) displacement control equipped with a 15 N load cell with Microtest SCM3000 95 software (Microtest, Madrid, Spain) (force resolution 0.001 N, position 0.001 mm, and time 0.1 s) to obtain force data (N), position (mm) and time (s). Data were recorded and exported at 0.5 s intervals to Microsoft Excel.

**Figure 5:**
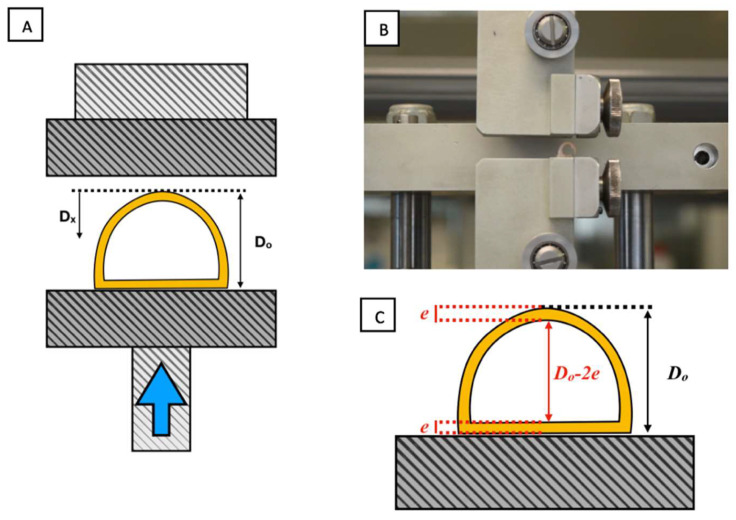
(**A**) Scheme depicting the trachea between the compression plates. Graphical definition of D_x_ (displaced distance) and D_o_ (initial anteroposterior diameter). (**B**) Trachea between the UTS plates. (**C**) Calculation of inner tracheal diameter from external diameter (*D_o_*) and trachea thickness (*e*).

The tracheas were placed with the membranous area resting on the lower plate, which was gradually raised towards the top plate at a constant speed of 5 mm·min^−1^ (Figure 5) [15,21].

To compare tracheal samples of different sizes, the force withstood per unit of length of the sample (*f* in N·mm^−1^) was calculated from the force value measured (*F*), and the sample length (*L*), according to the following the formula: *f* = *F*/*L*.

The UTS calculates the external anteroposterior initial diameter of the piece. However, the diameter of interest is the internal diameter (*D_i_*) as this determines the tracheal caliber. The *D_i_* is calculated by subtracting twice the measured mean thickness of the trachea from the external diameter *D_i_* = *D*_0_ − *2e* (Figure 5C).

The internal diameter of the trachea is reduced as the test proceeds and the percentage of tracheal occlusion (*O_l_*) can be computed by determining the ratio between the reduction of the internal diameter (equal to the jaw displacement, D_x_) and the initial internal diameter: *O_l_* = *D_x_*/*D_i_ 100*.

*f* vs. *O_l_* curves were drawn to characterise the specimens’ elastic properties. Occlusions of 25%, 50%, 75% and 100% were obtained and the force per length necessary for each degree of occlusion was determined. When the trachea is completely closed, the walls are compressed so that jaw displacement exceeds the initial internal diameter, which explains why the graph shows occlusions >100%.

The slope was calculated in N·mm^−1^ at each of these occlusion points in the graph. For occlusions of 25%, 50%, and 75%, the previous and subsequent five data points were taken (previous ten for 100% occlusion) using linear estimation. This calculated slope gives an approximation of the stiffness (*R*) of the trachea to radial compression in Mpa·mm and can be considered a measure of resistance to collapse.

We also obtained the area between the *f* versus *Occlusion* curve and the horizontal axis between 0% and 100% through the Riemann sum with approximation at the midpoint. The value obtained (in mJ·mm^−2^) indicates the energy per unit of surface area (*W*/*S*) needed to completely occlude the trachea.

### 2.5. Statistical Analysis

A total of eight fresh, decellularised rabbit tracheas were compared to eight native tracheas as controls. The study variables (except *f* and *R*) were analysed using multiple linear regression models. For the *f* and *R* variables, mixed linear regression models were applied. In these models, in addition to the variables of interest related to the treatment and condition of each trachea, the percentage occlusion was introduced as a monotonic effect and an independent term per trachea as a random factor. All models were adjusted by the Bayesian method using the R software program, v.3.5.3 R Core (R Foundation for Statistical Computing. 2019).

## 3. Results

### 3.1. Decellularisation

**Figure 6:**
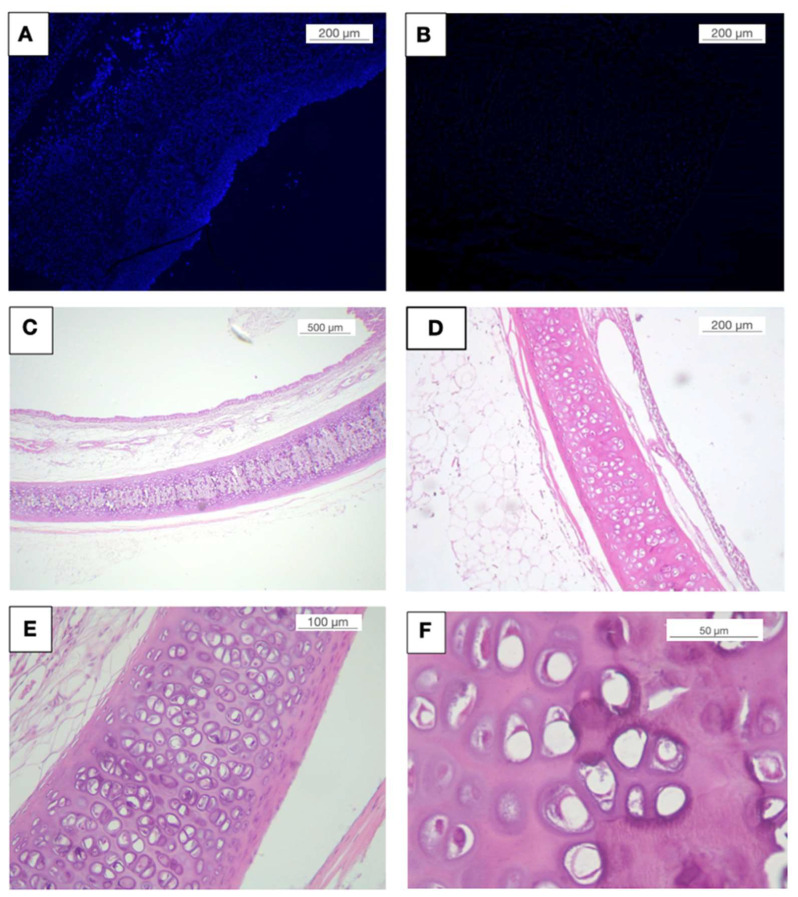
Histological analysis of decellularised tracheas. Fresh (**A**,**C**,**E**) and decellularised tracheas (**B**,**D**,**F**) were analyzed by DAPI (**A**,**B**) and by hematoxylin eosin (**C**–**F**). Cell removal in the decellularised tracheas was almost 100% compared to the fresh tracheas.

The tracheae were decellularised as described above. Cellular removal was evaluated by DAPI staining (Figure 6A,B). H-E staining showed a decellularised organ with minimal chondrocyte debris in cartilage (Figure 6C–F). DNA quantification did not detect values >50 ng or 200 pb in electrophoresis.

### 3.2. Tensile Tests

**Figure 7:**
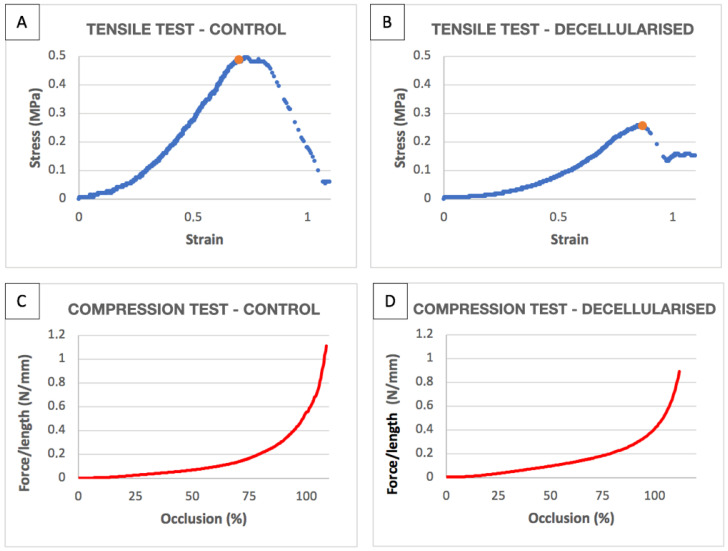
(**A**) Stress–strain graphs of tensile tests on a control trachea. (**B**) Stress–strain graphs of tensile tests on decellularised trachea. The orange dot marks the maximum or break point. (**C**) *f* curve by percentage occlusion of compression tests on a control trachea. (**D**) *f* curve by percentage occlusion of compression tests on a decellularised trachea.

The data obtained from the traction tests on the tracheas (controls and decellularised tracheas) are shown in Appendix A and in the Supplementary Materials (Video S1) and Figure 7A,B.

The decellularised tracheas showed a non-significant trend towards reduced *ε_max_*, (−0.204 mm CI [−0.407 and 0.005]) and *E* (−0.408 MPa CI [−688, −0.13] MPa) values. By contrast, the reduction in *σ_max_* was significantly lower (−246 MPa CI [−0.348, −0.145] MPa), as was *W*/*Vol* (-0.124 mJ·mm^−3^ CI [−0.195, −0.055] mJ·mm^−3^) in the decellularised tracheas compared to controls.

### 3.3. Compression Tests

The results of the compression tests are summarised in Appendix B and in the Supplementary Materials (Video S1) and Figure 6B,C.

No significant variations were observed in the *f* variable (0.001 N·mm^−1^ CI [−0.014, 0.008] N·mm^−1^), *R* (0.007 CI [−0.082, 0.07]), and *W*/*S* (−691 mJ·mm^−2^ CI [−1.419, −0.028] mJ·mm^−2^).

## 4. Discussion

The main challenge that any tracheal substitute must face in order to overcome the maximum resection length of 4.5 cm [3,5] are the same ones described by Belsey in the first ever report on a thoracic tracheal resection: lateral stiffness, elasticity, and longitudinal flexibility [17]. Although standardised histological studies have been developed to determine the presence of different cell types in organic samples [22], no standard approach to evaluating the biomechanical properties of the replacement—one of the most important features—has been developed to date.

Many of the experimental studies performed to date have used highly subjective techniques to evaluate the biomechanical properties of tracheal substitutes, such as compressing or folding the sample by hand, which does not provide objective results [23,24]. Although some studies have applied objective techniques, such as microscopic evaluation of the tissue, this is insufficient as it assesses only one part of the trachea (e.g., muscle, cartilage, mucosa, etc.) rather than the whole piece, which is the main point of interest in a tracheal substitute [25,26].

It is important to note that, while measurement standards such as the Standard Test Method for Tensile Properties of the American Society for Testing Materials have been established for inert materials, no such standards are available for bioengineering materials, which is particularly relevant for structurally complex organs like the trachea [25,27]. In this context, Jones et al. proposed a systematic method for measuring the biomechanical properties of the trachea. Those authors recommended using radial compression tests, similar to what we have proposed in the present study. However, in the model proposed by Jones et al., the strength exerted is considered the main criterion, but this fails to account for the dimensions (length and diameter) of the organ [15], which is why we have included these data points in our model. If we only consider the gross strength value, it would not be possible to compare data from tracheas of different sizes, since the strength value will vary according to the sample length. For this reason, we correlated strength with the length of the sample.

In our proposed model, we also recommend considering the occlusion of the internal lumen rather than of the external tracheal diameter, as the internal occlusion is the clinically relevant one [28]. Moreover, evaluation of the whole occlusion-force/length curve allows us to calculate *R* and *W*/*S*, not only at 50%, but at 25%, 50%, 75%, and 100%.

To reduce measurement variability in the tensile tests due to different stitch distances, we included the holes in the jaws, thus maintaining a constant inter-stitch distance in all the trials. We also adapted the type of suture to the size of the piece. The recommended type is between 3 and 4-0 in the adult trachea and 5-0 in pediatric cases [29,30]. A 2-0 suture seems to be too big for the anastomosis of small organs such as rabbit tracheas. Our proposal is to adapt the suture and anastomosis technique so that it can be performed exactly the same as it would be for an in vivo anastomosis, which is why we opted for a 6-0 monofilament running suture. To avoid possible systematic bias due to the chosen suture and jaws (which would also make it impossible to compare different studies), it is vitally important to calculate the deformation of the jaw-suture assembly for each unit of force and then subtract this from each measure.

The data obtained from the UTS are related to the tracheal dimensions, using stress and not force for the calculations, as the effect of the force applied on the organ logically varies according to its surface area. For deformation, we refer to the initial tracheal measure, thus handling the strain. This allows for a proper comparison of tracheas, regardless of the size.

The determination of Young’s modulus in the study by Jones et al. is described somewhat arbitrarily as the line of cut between a parallel line 0.22 mm to the right of the linear zone of the curve with the curve itself [15]. By contrast, in our model, we mathematically defined both the breakage point (the point at which tension drops by >1% without recovering within the next 0.8 s, or any reduction >10%), and Young’s modulus (slope of the last linear segment of the curve before reaching the proportional limit in which it grew compared to the previous one), which again avoids variability within these measures.

The results obtained in this study for the decellularised tracheas are consistent with previous reports showing a loss in biomechanical properties versus the native trachea. These are in fact reduced only in our *σ_max_* y *W*/*Vol* model in tensile tests.

The main limitations of the present study are those related to the construction of the jaw, since it was built to fit the size and shape of a rabbit trachea. When applied to tracheas of different origins (and thus different sizes and proportions), the jaws must be adapted to that shape.

## 5. Conclusions

In order to ensure proper airway functioning, it is of paramount importance that the biomechanical properties of the substitute airway are as similar as possible to those of the native trachea. Consequently, it is essential to systematically evaluate the biomechanical properties of replacement airways to compare them to native tracheas, which would then allow us to select the best scaffold option. Our strategy ensures a biomechanically appropriate substitute, which maintains its biomechanical characteristics. Moreover, our proposed testing protocol is systematic, repeatable, and reproducible. The implementation of a systematic method to biomechanically evaluate tracheal replacements would facilitate the proper and objective comparison of different strategies, regardless of the size or shape of the substitute.

## Data Availability

Not Applicable.

## Supplementary Materials

The following are available online at https://www.mdpi.com/article/10.3390/biom11101461/s1, Video S1: Biomechanical tensile and compression tests.

## Appendix A

**Table A1:** Tensile test of control and decellularised tracheas.

	$\sigma_{max}\;\left(MPa\right)$	$\varepsilon_{max}$	WVol(mJmm3)	E(MPa)	R^2^
**Control 1**	0.40420303	0.90215056	0.12909439	0.953563904	0.99875489
**Control 2**	0.54856126	0.81976858	0.15110825	1.368955241	0.99938136
**Control 3**	0.38803491	0.93763317	0.12716517	0.83060708	0.99864644
**Control 4**	0.63815134	1.01073746	0.30837771	1.16485761	0.99327878
**Control 5**	0.48841058	0.69674901	0.15157978	1.08214574	0.99917287
**Decellularised 1**	0.23126649	0.8447467	0.08295248	0.450701404	0.997401629
**Decellularised 2**	0.07906587	0.60231885	0.02377653	0.194237888	0.995152737
**Decellularised 3**	0.25704701	0.86614847	0.08670318	0.560490726	0.99803033
**Decellularised 4**	0.45882506	0.58685413	0.12242781	1.54950156	0.998076727

$\sigma_{max}$: Maximum stress; $\varepsilon_{max}$: Maximum strain; WVol: energy per unit of volume; E: Young modulus; R2: Correlation coefficient

## Appendix B

**Table A2:** Compression tests on control and decellularised tracheas.

		f(Nmm)	ℛ(Mpa·mm)	R^2^	WS(mJmm2)
**Control 1**	25%	0.025101336	0.1638327	0.997012177	0.11763228
	50%	0.068802392	0.222956354	0.998417427	
	75%	0.16695744	0.719024065	0.998370916	
	100%	0.55047896	2.884581423	0.986746954	
**Control 2**	25%	0.01263868	0.07017383	0.91366111	0.06175561
	50%	0.03600847	0.11793468	0.98844924	
	75%	0.08392836	0.36330023	0.99356025	
	100%	0.29732534	2.4991525	0.99724707	
**Control 3**	25%	0.02655163	0.12965658	0.97408101	0.09201494
	50%	0.05756974	0.15144803	0.98578378	
	75%	0.1216738	0.47044318	0.99818636	
	100%	0.4415049	3.42037414	0.99662314	
**Control 4**	25%	0.02830126	0.11819036	0.98279867	0.0896916
	50%	0.05960658	0.14729238	0.98500466	
	75%	0.12236075	0.40243118	0.99626884	
	100%	0.4391475	4.46150808	0.99538744	
**Control 5**	25%	0.01756126	0.05047939	0.99219239	0.04554423
	50%	0.03210593	0.06333302	0.99822932	
	75%	0.06119527	0.18608305	0.99595152	
	100%	0.16047853	0.98869009	0.98834538	
**Decellularised 1**	25%	0.00918811	0.01024683	0.90599479	0.01422099
	50%	0.01152998	0.01038917	0.96669188	
	75%	0.01660403	0.04111375	0.98696429	
	100%	0.04080339	0.2084726	0.98459284	
**Decellularised 2**	25%	0.00520851	0.01083867	0.95625405	0.00895111
	50%	0.0082675	0.01479881	0.97195543	
	75%	0.01204025	0.01638842	0.98380543	
	100%	0.01815821	0.04074716	0.98841792	
**Decellularised 3**	25%	0.00699906	0.01305482	0.9419434	0.01376554
	50%	0.01157826	0.01950127	0.97465106	
	75%	0.01832657	0.03969066	0.99386615	
	100%	0.0390535	0.24461473	0.97000712	
**Decellularised 4**	25%	0.03447359	0.23078059	0.99953159	0.12094272
	50%	0.09601747	0.27874796	0.99952229	
	75%	0.18117175	0.45357536	0.99904677	
	100%	0.40507942	1.9143551	0.99285479	
